# Extraction and Determination of Cyproheptadine in Human Urine by DLLME-HPLC Method

**Published:** 2013

**Authors:** Mehdi Maham, Vahid Kiarostami, Syed Waqif-Husain, Parviz Abroomand-Azar, Mohammad Saber Tehrani, Malihe Khoeini Sharifabadi, Hossein Afrouzi, MahmoudReza Shapouri, Rouhollah Karami-Osboo

**Affiliations:** a *Department of Chemistry, Science and Research Branch, Islamic Azad University ,P.O.Box 14515- 775, Poonak Hesarak, Tehran, Iran. *; b *Department of Chemistry, North Tehran Branch, Islamic Azad University, Tehran, Iran. *; c *Quality Control laboratory, DarouPakhsh Mfg. Co., Tehran, Iran. *; d *Students Research Committee, Shiraz University of Medical Sciences, Shiraz, Iran.*

**Keywords:** Antihistamine, Cyproheptadine, DLLME, HPLC, Human urine

## Abstract

Novel dispersive liquid-liquid microextraction (DLLME), coupled with high performance liquid chromatography with photodiode array detection (HPLC-DAD) has been applied for the extraction and determination of cyproheptadine (CPH), an antihistamine, in human urine samples. In this method, 0.6 mL of acetonitrile (disperser solvent) containing 30 μL of carbon tetrachloride (extraction solvent) was rapidly injected by a syringe into 5 mL urine sample. After centrifugation, the sedimented phase containing enriched analyte was dissolved in acetonitrile and an aliquot of this solution injected into the HPLC system for analysis. Development of DLLME procedure includes optimization of some important parameters such as kind and volume of extraction and disperser solvent, pH and salt addition. The proposed method has good linearity in the range of 0.02-4.5 μg mL^-1 ^and low detection limit (13.1 ng mL^-1^). The repeatability of the method, expressed as relative standard deviation was 4.9% (n = 3). This method has also been applied to the analysis of real urine samples with satisfactory relative recoveries in the range of 91.6-101.0%.

## Introduction

Antihistamines are a class of pharmaceutical compounds, which act by stimulating the histamine action in the H_1_receptors, antagonizing most of the smooth muscles. Antihistamines are used to relieve or prevent the symptoms of hay fever and other allergies. Antihistamine such as cyproheptadine (CPH [4-(5H-diben-zo [*a*, *d*] cyclohepten-5- ylidene)-1-methylpiperidine]) are known to treat a variety of allergic disorders with H_1_- antihistamine properties (1, 2).

Using effective methods for extraction of pharmaceutical compounds in biological matrices is of great importance. Different methods such as liquid-liquid extraction (LLE) (3, 4) and solid phase extraction (SPE) (2, 5) have been used for separation and preconcentration of some drugs. Liquid phase microextraction (LPME) technique has been developed as an alternative to the classical LLE and SPE techniques (6-10). Recently Rezaee *et* *al. *(11) have reported a new dispersive liquidliquid microextraction (DLLME) technique. In DLLME methodology, a binary mixture of a water-immiscible organic solvent (extractant) and a water-miscible organic solvent (disperser) is rapidly injected into the aqueous sample containing the analyte. Consequently, a cloudy solution (high turbulence) forms, which consists of fine droplets with a quite large surface area and the analyte is freely extracted into the fine droplets of extractant dispersed into the aqueous solution. After centrifugation of the cloudy solution, a sedimented phase is settled at the bottom of a conical test tube and analyzed with an appropriate analytical technique.

 The application of DLLME has been increased in trace analysis (12, 13). Xiong *et al. *(14) presented a DLLME procedure for extraction of three psychotropic drugs in urine samples. Sarafraz Yazdi *et al*. (15) have applied DLLME for separation of amitriptyline and nortriptyline in blood plasma. Several analytical methods have been used for the determination of CPH, such as highperformance liquid chromatography (HPLC) (16), liquid chromatographic-tandem mass spectrometric (LC-MS/MS) (17, 18) and capillary electrophoresis (2, 19). In the present work, an improved DLLME method has been developed for pre-concentration of CPH in the urine samples. The analyte is isolated from the urine matrix using DLLME without dilution and subsequently analyzed by HPLC with photodiode array detection (DAD).

## Experimental

*Reagents and materials*

**Figure 1:**
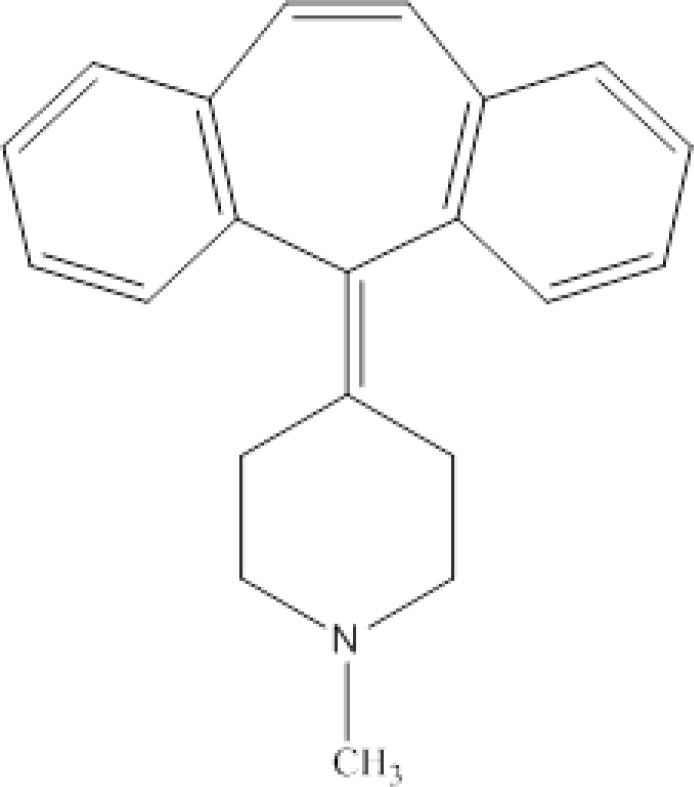
Chemical structure of cyproheptadine

Cyproheptadine was obtained from United States Pharmacopeia (USP). Water and acetonitrile were of HPLC grade and the other chemicals used in this study were of analytical grade and obtained from E. Merck (Germany). Stock solution of CPH (10 mg L^-1^) was prepared by dissolving the appropriate amount of the corresponding pure salt in acetonitrile and stored at 4°C. The working standard solutions were prepared by serial spiking of drug free urine samples with the standard solution. Drug free urine samples were collected from healthy adult not exposed to any drug for at least 2 months. Real urine samples collected from female patient under treatment. Chemical structure of CPH is shown in Figure 1.

*Urine Sample preparation*

Drug free urine samples were spiked with CPH and made alkaline using sodium hydroxide. The solution was centrifuged for 10 min at 4000 rpm and a white solid lipid sedimented in the bottom of the conical test tube, probably due to the co-sedimentation of matrixes (such as urea and uric acid) in urine at high pH values. The resulting supernatant liquid was then subjected to the DLLME process.

*Extraction procedure*

Five mL aliquot of the pretreated urine sample was placed in a 10 mL conical test tube. Thirty μL CCl_4_ and 0.6 mL acetonitrile (optimum values) was injected rapidly into the sample using a 1 mL syringe. At this step, a cloudy solution formed in the test tube and the analyte in urine sample was extracted into the fine droplets of CCl_4_. Then, the mixture was centrifuged for 10 min at 4000 rpm. After centrifuging, the dispersed fine droplets of extractant were sedimented and whitish interface was observed between the settled drop of CCl_4_ and the upper aqueous phase in the test tube. The upper aqueous solution was removed with a syringe and the residual phase was dissolved in 300 μL acetonitrile. Finally, 20 μL of the extract was injected into the HPLC system for analysis.

*Instrument*

The chromatographic analyses were performed using a HPLC system (Waters) equipped with two 515 HPLC pumps (Waters) and a photodiode array detector (Waters 996). A reversed-phase L_7_-C_8_ symmetry column (250 × 4.6 mm I.D., particle size 5μm) was used for separation at ambient temperature. A mixture of ammonium acetate (0.03 M, pH = 5.5) and acetonitrile (50:50 v/v) was used as the mobile phase at a flow rate of 1 mL min^-1^ in isocratic elution mode. The injection volume was 20 μL for all the solutions and the detection was performed at the wavelength of 265 nm. A centrifuge model Clements GS200 was used for separation of the extraction phase.

## Results and Discussion

To obtain high extraction efficiency, it is necessary to investigate the effect of all parameters that may possibly influence the performance of DLLME. These parameters include the type and the volume of the extraction and the disperser solvents, the salt addition and pH.

*Effect of the extraction solvent type*

The extraction solvent should be carefully chosen. In the classical DLLME technique, the selection of an appropriate extraction solvent is of high importance since the target analytes should be efficiently desorbed and the remaining matrix components should be retained in the matrix. Hence, the extraction solvent should have a higher density than water, extraction capability of the interested compounds and low solubility in water. It is important that the selected extraction organic solvent for DLLME method be compatible with the HPLC mobile phase. However, halogenated hydrocarbons usually selected as extracting solvents in DLLME, are not compatible with the reversephase- HPLC mobile phase because of their high density and an extra step is required to dissolve them in compatible organic phase or mobile phase before final analysis. Carbon tetrachloride (CCl_4_), chlorobenzene (C_6_H_5_Cl) and dichloromethane (CH_2_Cl_2_) were compared as extraction solvents. In the beginning, a series of experiments were performed by using 600 μL acetonitrile as the disperser solvent and 20 μL of several kinds of extraction solvents for optimization of extraction solvent type. The results show that, in the case of CH_2_Cl_2_, a twophase system was not observed because of the high solubility of CH_2_Cl_2_ in aqueous solution (15, 20, 21). The best performance was obtained when CCl_4_ was used as the extraction solvent. Thus, CCl_4_ was selected as the extraction solvent in the following experiments.

*Effect of the disperser solvent type*

**Figure 2:**
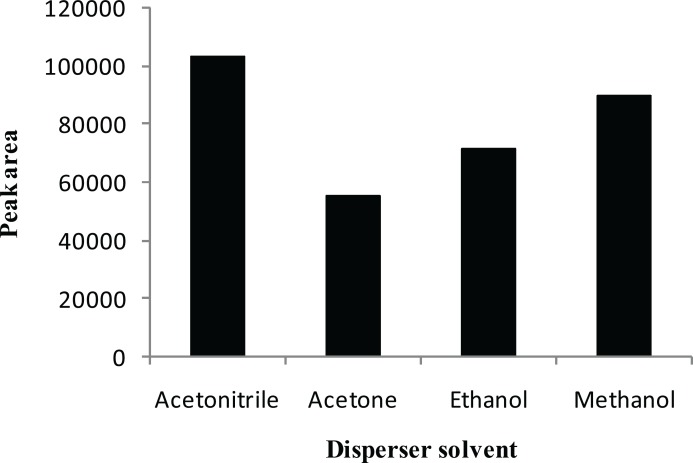
Effect of the disperser solvent type on the extraction efficiency

The main criterion for selection of the disperser solvent is its miscibility in the organic (extraction solvent) and aqueous (sample solution) phases. In this study, the suitability of acetonitrile, acetone, methanol and ethanol, which have the capabilities listed above, was investigated with a series of sample solutions by using 0.6 mL of each disperser solvent containing 20.0 μL CCl_4_. As shown in Figure 2, acetonitrile has the highest efficiency compared with acetone, methanol and ethanol. Therefore, acetonitrile was selected as the disperser solvent in the subsequent experiments.

*Effect of the extraction solvent volume*

**Figure 3:**
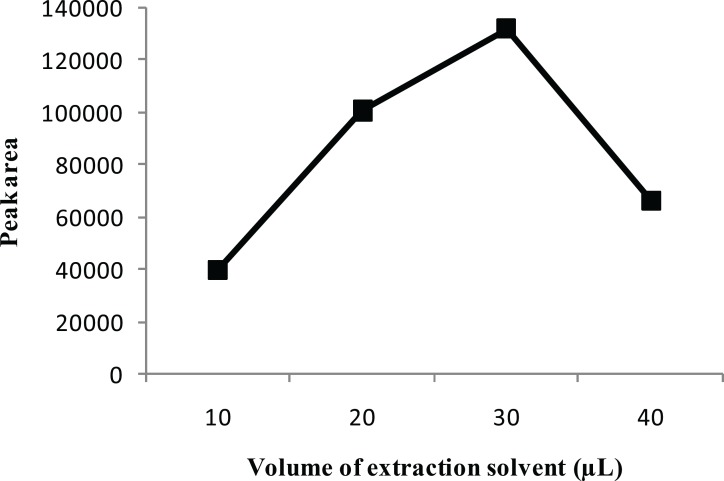
Effect of the volume of extraction solvent on the extraction efficiency. Separation and Determination of Cyproheptadine in Human Urine by DLLME-HPLC Method, Mehdi Maham

To examine the effect of the extraction solvent volume, the volume of CCl_4_ was varied in the range of 10-40 μL, with other experimental conditions being constant. Figure 3 shows that the extraction recovery increased by increasing the volume of CCl_4_ to 30 μL. However, a reduction in the extraction recovery for CPH occurred when the volume of CCl_4_ exceeded 30 μL. This is probably due to the variation of the volume ratio between the disperser and the extraction organic solvents. The decreased ratio lowers the amount of droplets formation available for extraction, thereby lowering the extraction efficiency. Based on the experimental results, 30 μL of CCl_4_ was chosen as the optimal volume for the extraction solvent.

*Effect of the disperser solvent volume*

**Figure 4:**
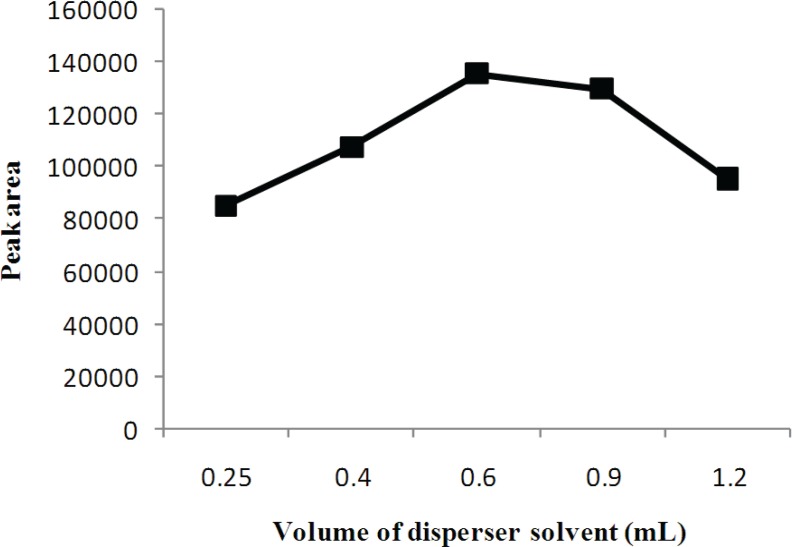
Effect of the volume of disperser solvent on the extraction efficiency. Separation and Determination of Cyproheptadine in Human Urine by DLLME-HPLC Method, Mehdi Maham.

Disperser solvent volume is important to make extraction solvent form very fine droplets, which directly affect the extraction efficiency. The influence of the volume of disperser solvent was investigated by using 0.25, 0.4, 0.6, 0.9 and 1.2 mL volumes. According to Figure 4, the extraction efficiency increases by increasing the volume of acetonitrile to 0.6 mL and then decreases at volumes over 0.6 mL. The increase in extraction efficiency was attributed to the much finer droplets and larger surface area of extraction solvent, obtained by increased acetonitrile volume. Decrease in extraction efficiency was related to the increase in solubility of the analyte in the aqueous phase. Therefore, based on the obtained results, 0.6 mL of acetonitrile was chosen as optimum volume for disperser solvent.

*Effect of pH*

**Figure 5:**
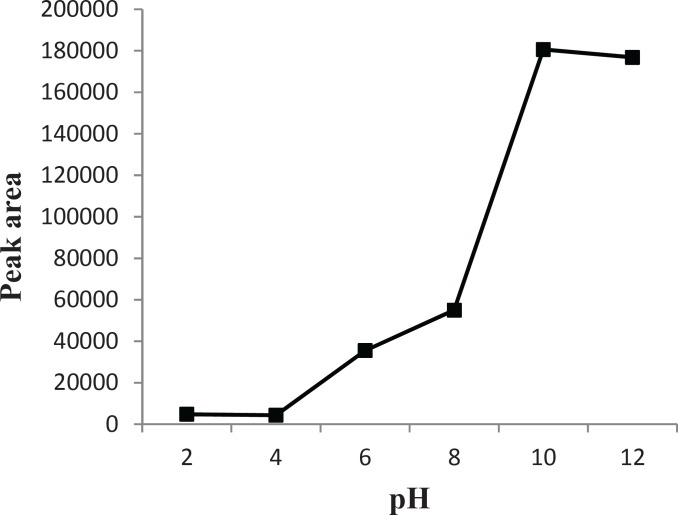
Effect of the pH values on the extraction efficiency. Separation and Determination of Cyproheptadine in Human Urine by DLLME-HPLC Method, Mehdi Maham

When the pH changes, the acid-base equilibrium for compounds containing functional groups significantly shifts towards neutral or ionic forms. Thus, their solubility in the sample solution enhances or reduces. In DLLME, for basic analytes [according to the literature (2), the p*K*_a_ value of CPH is 9.3] the pH in the sample should be higher than the p*K*_a_ values of analytes. This way, most of the analyte species are uncharged and readily extracted into organic phase. This is a requirement for optimal partitioning and therefore enrichment in the organic phase. The pH of sample solutions was optimized over the range of 2-12. The results (Figure 5) show that the extraction efficiency significantly increased when pH was higher than p*K*_a_ value of the analyte. The results proved that the solution pH was a critical factor, which affects the extraction recoveries of CPH in urine samples. Hence, pH = 10 was used as the optimum value in the following experiments.

*Effect of salt addition*

**Figure 6:**
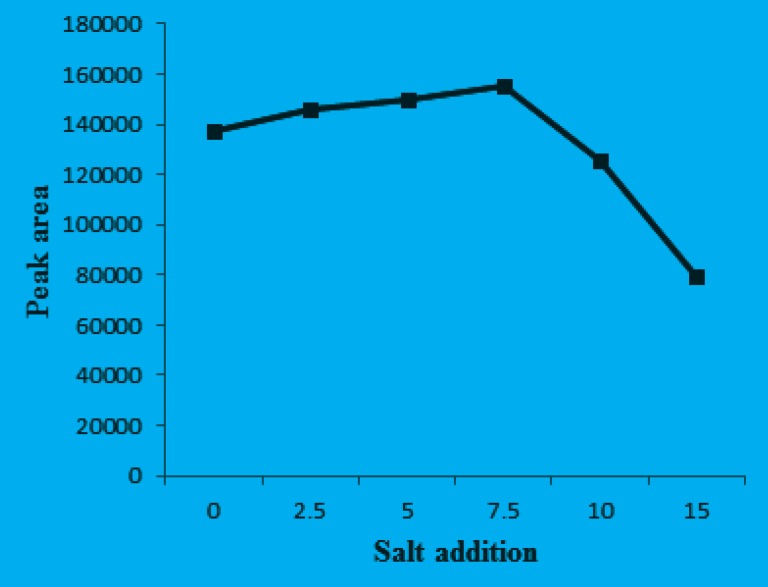
Effect of the salt addition on the extraction efficiency. Separation and Determination of Cyproheptadine in Human Urine by DLLME-HPLC Method, Mehdi Maham.

In this study, the salt effect on performance of the DLLME was evaluated by increasing sodium chloride concentration in the sample solution from 0% to 15% (m/v). The other DLLME parameters were used according to the obtained optimum values and the results are shown in Figure 6. 

It is evident that the extraction efficiency increases with the addition of sodium chloride over the range of 0-7.5% and then decreases with the further salt addition. These results could be explained from several aspects. First, extraction efficiency increased due to the salting out effect, whereby water molecules from hydration spheres around the ionic salt molecules reduce the concentration of water available to dissolve the analyte molecules and then decrease the solubility of the target analyte in the aqueous phase (22), thus enhancing transfer of analyte into the organic phase. Secondly, decrease in the extraction efficiency is due to the increased viscosity of the solution, thus reducing the rate of diffusion of the target analyte into the extraction solvent (23). These observations showed the possibility of using this method for separation of CPH from saline solution up to 7.5%. The optimum DLLME conditions were as follows: 30.0 μL CCl_4_ as extraction solvent; 0.6 mL acetonitrile as disperser solvent; pH = 10 and 7.5% salt addition. 

*Quantitative analysis*

A Calibration curve was drawn utilizing spiking levels of drug in drug free urine samples. For each level, three replicate extractions were performed at optimal conditions. Under the optimized conditions, the calibration curve was linear in the range of 0.02-4.5 μg mL^-1^ with a good correlation coefficient (0.9983). The preconcentration factor is defined as the ratio of calibration curve slope with and without preconcentration and was 16.2. The repeatability, expressed as relative standard deviation (RSD), was 4.9%. The limit of detection (LOD), based on S/N = 3, and the limit of quantification (LOQ), based on S/N = 10, were 13.1 and 20.69 ng mL^-1^, respectively.

*Real urine sample analysis*

The matrix effects on the extraction were also evaluated by investigating the applicability and accuracy of the proposed method to determine CPH concentrations in real urine samples obtained from female patient under treatment. Female patient received a single oral dose of cyproheptadine tablet and the urine samples were collected 12 h after the administration and stored in PTFE flasks at - 20°C prior to analysis. The concentration of CPH in the patient urine samples was 0.032 ± 0.002 μg mL^-1^. These samples were spiked with CPH standards at different concentration levels to assess matrix effects and analyzed by HPLC-DAD. The results (Table 1) show that relative recoveries (expressed as the mean percentage between the amounts found and added) were in the range of 91.6- 101.0%. Good agreement was obtained between the added and found analyte values using the recommended procedure. This indicated that the presence of major endogenous components and drug metabolites in urine samples did not influence the performance of this developed method. Therefore, the DLLME-HPLC-DAD method is effective for quantitative analysis of CPH in urine samples.

**Table 1:** Relative recoveries of spiked CPH in patient urine samples under the treatment^A^

**Antihistamine**	**Initial concentration** **mean ± SD** ^B^ ** (μg mL** ^-1^ **)**	**Concentration added** **(μg mL** ^-1^ **)**	**Concentration determined** **mean ± SD** ^B^ ** (μg mL** ^-1^ **)**	**Relative recovery (%)**
CPH	0.032 ± 0.002	0.08	0.113 ± 0.004	101.0
CPH	0.032 ± 0.002	0.12	0.144 ± 0.006	93.2
CPH	0.032 ± 0.002	0.18	0.197 ± 0.007	91.6

^A^ Extraction conditions; extraction solvent and its volume = 30 μL CCl4; disperser solvent and its volume: 0.6 mL acetonitrile; pH value = 10; ionic strength = 7.5 %. ^B^ SD, standard deviation (n = 3

*Comparison with SPE method*

A comparison of the present method with SPE method for the pre-concentration of CPH in urine samples is given in Table 2. It is evident that the present method is an environmentally benign sample preparation method because of its consumption of very small amounts of organic solvents and consequent production of a lower amount of organic waste. Thus, minimum exposure to toxic organic solvents makes it safe for the analyst. The LOD and LOQ obtained from the present method are lower than those from SPE approach used in combination with HPLC. Additionally, this method has some further advantages such as consumption of small amount of sample, simplicity of operation and low analysis cost. These characteristics are of great interest for the routine laboratories and the method developed in this work is recommended as a suitable alternative to traditional methods in the analysis of pharmaceutical compounds.

**Table 2:** Comparison of the proposed method with SPE method

**Antihistamine**	**Method**	**Sample** **consumption (mL)**	**Extraction solvent** **volume (μL)**	**LODs(ngmL** ^-1^ **)**	**LOQs(ngm** ^-1^ **)**	**Linear range** **(ng mL** ^-1^ **)**	**Ref.**
CPH	SPE-HPLC-UV^A^	1	6000	15.0	50	53-530	(16)
CPH	DLLME-HPLC-DAD	5	30	13.1	20.69	20.69-4500	This work

^A^ UV, ultraviolet

## Conclusion

In the present study, a new optimized method of DLLME combined with HPLC-DAD has been developed for determination of CPH in urine samples. The analyte is pre-concentrated from the urine matrix without any dilution or decrease in sensitivity. The results show that the developed method has good repeatability, low LOD and LOQ, minimum consumption of toxic organic solvents and low cost. In summary, this new sample preparation technique can be widely used in many areas of pharmaceutical and environmental analysis, such as more complicated matrices (biological samples), as a rapid, simple and inexpensive method.
